# Apigenin inhibits TNFα/IL-1α-induced CCL2 release through IKBK-epsilon signaling in MDA-MB-231 human breast cancer cells

**DOI:** 10.1371/journal.pone.0175558

**Published:** 2017-04-25

**Authors:** David Bauer, Natalie Redmon, Elizabeth Mazzio, Karam F. Soliman

**Affiliations:** College of Pharmacy and Pharmaceutical Sciences, Florida A & M University, Tallahassee, Florida, United States of America; University of South Alabama Mitchell Cancer Institute, UNITED STATES

## Abstract

Mortality associated with breast cancer is attributable to aggressive metastasis, to which TNFα plays a central orchestrating role. TNFα acts on breast tumor TNF receptors evoking the release of chemotactic proteins (e.g. MCP-1/CCL2). These proteins direct inward infiltration/migration of tumor-associated macrophages (TAMs), tumor-associated neutrophils (TANs), myeloid-derived suppressor cells (MDSCs), T-regulatory cells (Tregs), T helper IL-17-producing cells (Th17s), metastasis-associated macrophages (MAMs) and cancer-associated fibroblasts (CAFs). Tumor embedded infiltrates collectively enable immune evasion, tumor growth, angiogenesis, and metastasis. In the current study, we investigate the potential of apigenin, a known anti-inflammatory constituent of parsley, to downregulate TNFα mediated release of chemokines from human triple-negative cells (MDA-MB-231 cells). The results show that TNFα stimulation leads to large rise of CCL2, granulocyte macrophage colony-stimulating factor (GMCSF), IL-1α and IL-6, all suppressed by apigenin. While many aspects of the transcriptome for NFkB signaling were evaluated, the data show signaling patterns associated with CCL2 were blocked by apigenin and mediated through suppressed mRNA and protein synthesis of IKBKe. Moreover, the data show that the attenuation of CCL2 by apigenin in the presence TNFα paralleled the suppression of phosphorylated extracellular signal-regulated kinase 1 (ERK 1/ 2). In summary, the obtained findings suggest that there exists a TNFα evoked release of CCL2 and other LSP recruiting cytokines from human breast cancer cells, which can be attenuated by apigenin.

## Introduction

Breast cancer is a leading cause of death in women worldwide, despite medical advances in surgical techniques, radiation and chemotherapy. There is a continued need to identify preventive and adjunctive chemosensitization agents in the management of breast cancer. Apigenin (4',5,7-trihydroxyflavone) is naturally occurring bioactive compound (NOBC) found in parsley, garlic, and celery. The biological relevance of apigenin in management of breast cancer is evidenced by the growing pool of research (*in vitro* and xenograft tumor models) demonstrating its capacity to inhibit tumor promoting enzymes [1,2], overcome chemoresistance [3,4] and/or multi-drug resistance [5], prevent angiogenesis [6] metastasis and lessen the growth of aggressive breast cancers [7]. There is also a plethora of research showing apigenin as a cytostatic agent with capacity to attenuate clonogenic survival in a wide range of breast cancer cells; MDA-MB-231 [8], SKBR3 [9,10], Hs578T, MCF-7 [11], MDA-MB-453 [12], MDA-MB-468 Cells [13,14], T47D [15] and BT-474 Cells [16]. Biological targets of apigenin relevant to tumor potential are known to involve MAPK/NFkappaB, phospho-JAK1/ STAT3 signaling [17], VEGF, aromatase, proteasomal processes, fatty acid synthase [18–21] and the Fas-associated protein with death domain (FADD) [22].

Despite surmounting evidentiary support for apigenin in tumor suppression, there is lack of research regarding its influence on the tumor microenvironment in response to pro-inflammatory cytokines, specifically TNFα. TNFα is present in high concentrations throughout the breast tumor/ stroma milieu, and upon activation of TNFα receptors, can trigger a powerful perpetual cascade of NF-κB activation [23,24]. epithelial-to-mesenchymal transition [25] and sustained release of diverse chemokines (i.e., CCL2/CCL5) [26]. Chemokines in turn ultimately trigger inward migration of a large number of leukocyte sub-populations (LSPs) bearing CCR2 / CCR5 receptors that embed into the tumor, and further drive tumor aggression and stem cell survival. [27,28] Tumor promoting LSPs are known to include tumor-associated macrophages (TAMs) [29], myeloid-derived suppressor cells (MDSCs) [30], tumor-associated neutrophils (TANs)[31,32], T-regulatory (Tregs) [33] metastasis-associated macrophages (MAMs), T helper IL-17-producing cells (Th17) cells and cancer-associated fibroblasts (CAFs) [34].

Thus, given the dynamic control of diverse chemokines as key trafficking molecules released by tumor cells in response to TNFα capable of driving LSP recruitment [35–38], here we investigated the effects of apigenin on TNFα mediated chemokine release in triple-negative breast cancer (TNBC) cells.

## Results

**Fig 1:**
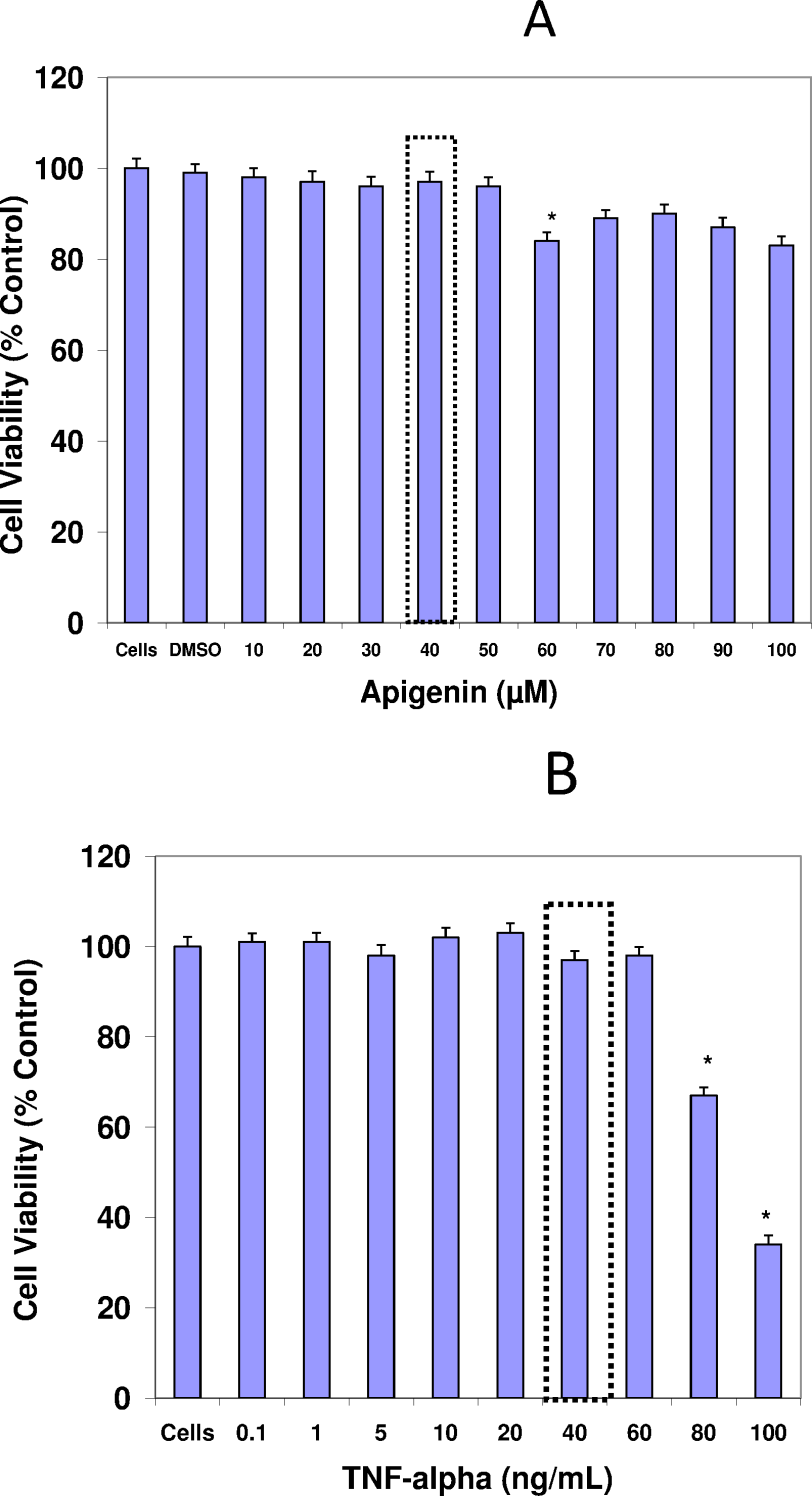
**The effect of apigenin (A) and TNFα (B) on cell viability of MDA-MB-231 cells at 5% CO**_**2**_**/Atm for 24 Hr.** The data are presented as viability (% Ctrl), Mean ± S.E.M. n = 4. The significance of differences from the Ctrl and were determined by a one-way ANOVA, with a Tukey post hoc test. *p<0.05.

**Fig 2:**
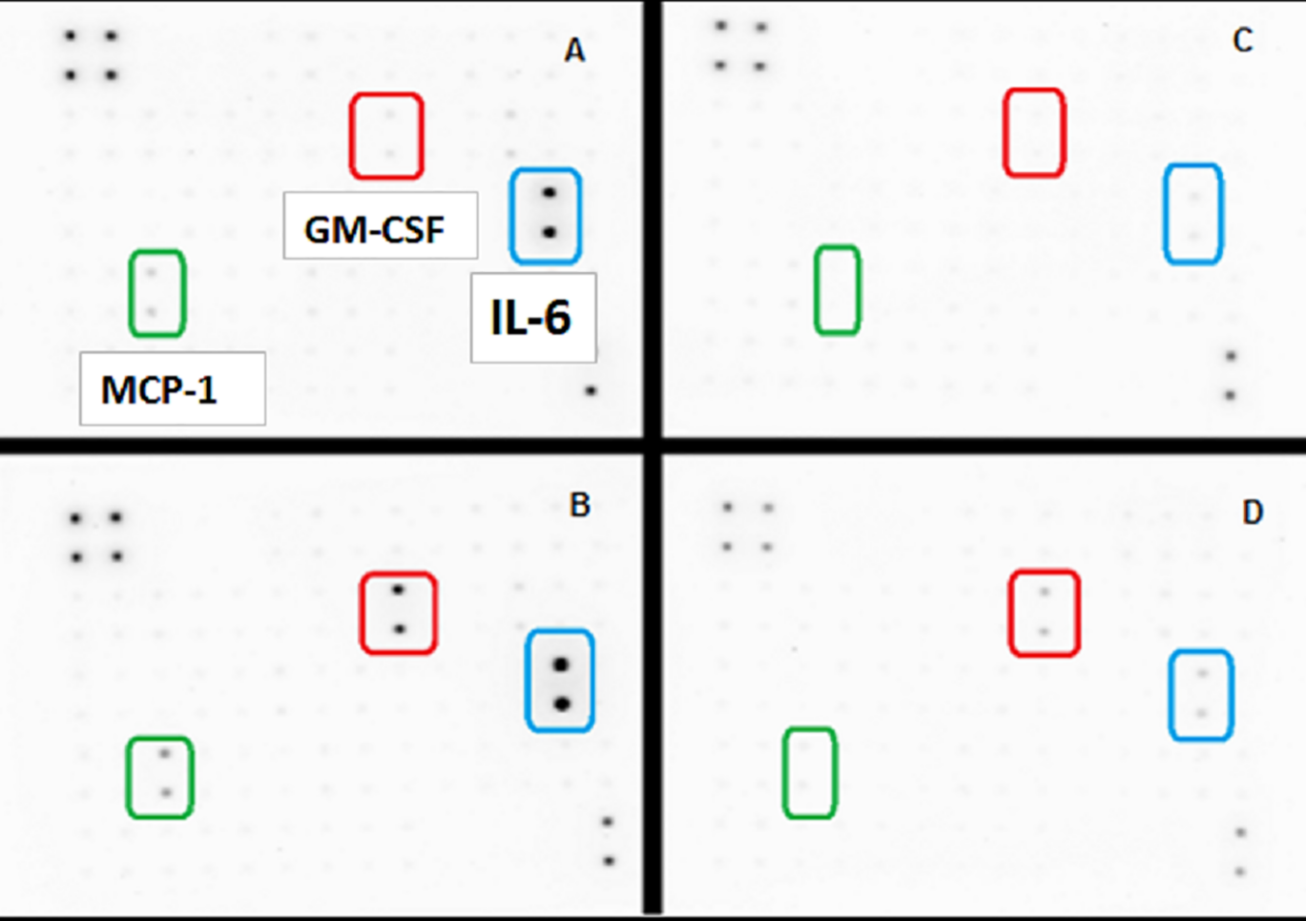
**(A-D). The release of cytokines as assessed using Human Cytokine Array C1000 membranes from MDA-MB-231 cells ± TNFα ± Apigenin. [A]** Ctrls, **[B]** TNFα-treated (40ng/mL) **[C]** Apigenin (40μM) and **[D]** TNFα-treated (40ng/mL) + Apigenin (40μM) co-treated cells.

**Fig 3:**
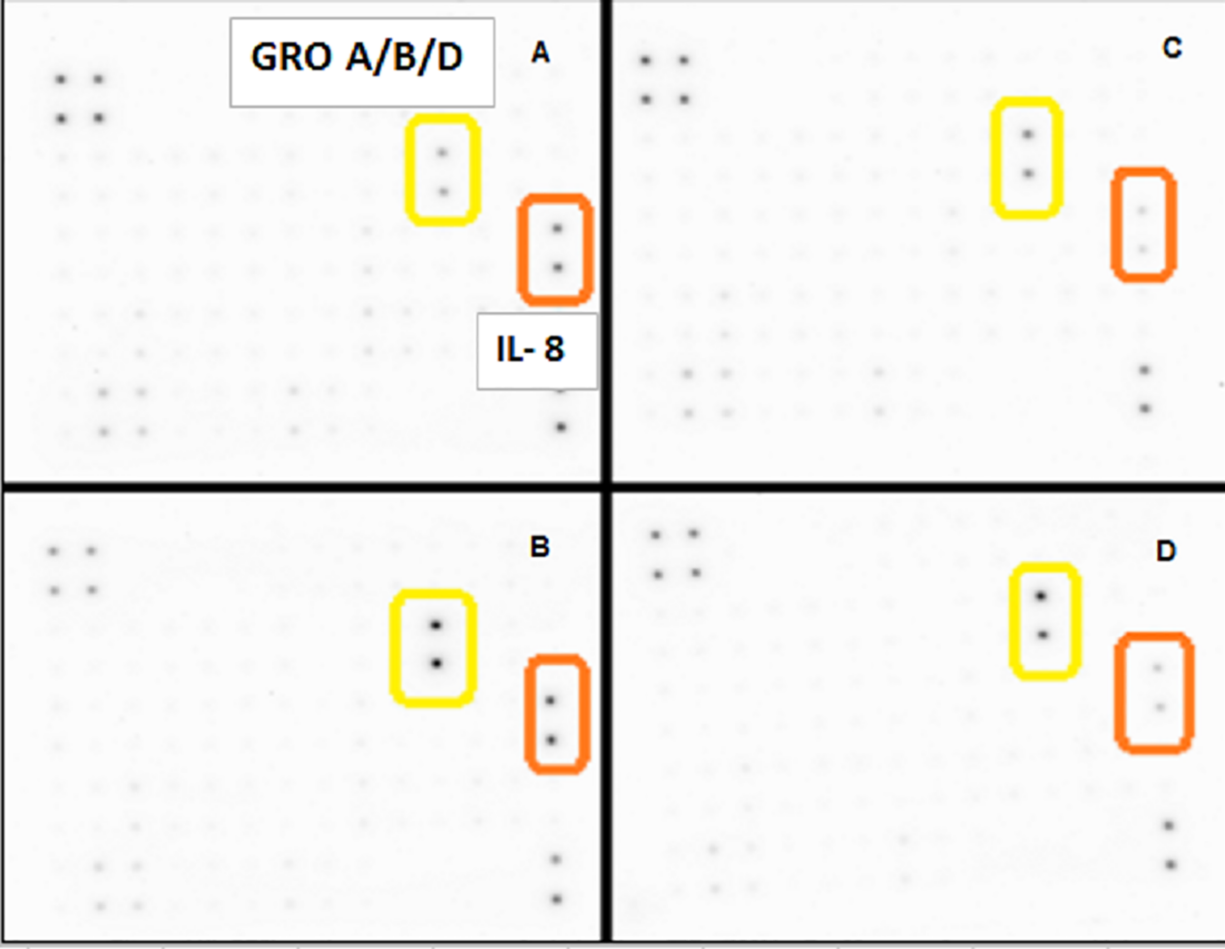
**(A-D). The release of cytokines as assessed using Human Cytokine Array C1000 membranes from MDA-MB-231 cells ± TNFα ± Apigenin. [A]**Ctrls, **[B]**TNFα-treated (40ng/mL) **[C]**Apigenin (40μM) and **[D]**TNFα-treated (40ng/mL) + Apigenin (40μM) co-treated cells. The data present membrane spot densities (top) and manufacturer’s grid layout (bottom).

**Fig 4:**
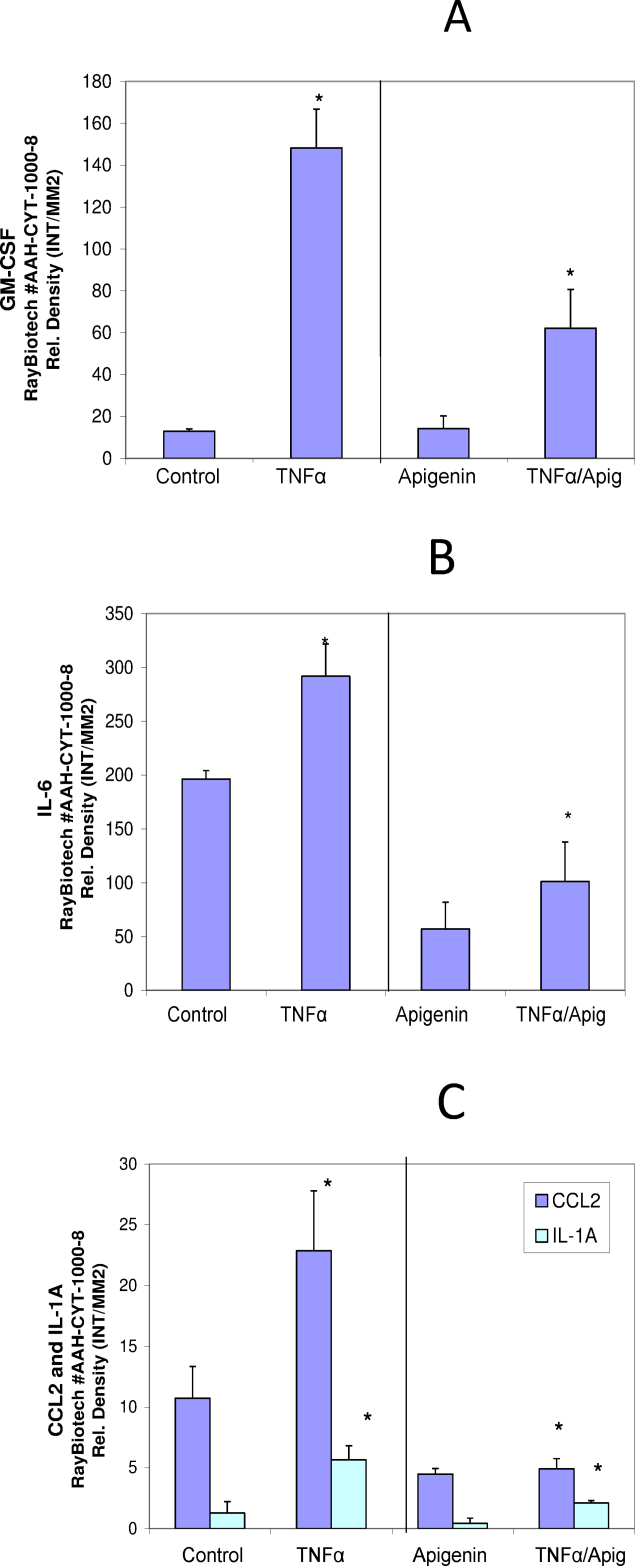
**The effect of apigenin on TNFα mediated changes in GM-CSF (A) IL-6 (B), CCL2 and IL-1α (C) in MDA-MB-231 cells**. The data represent the densitometry values (expressed as intensity per square millimeter) (INT/MM2) per spot as Mean ± S.E.M. n = 3. The significance of differences between the Ctrl and TNFα groups and TNFα vs. TNFα + Apigenin were determined by a Students t-test *p<0.05.

**Fig 5:**
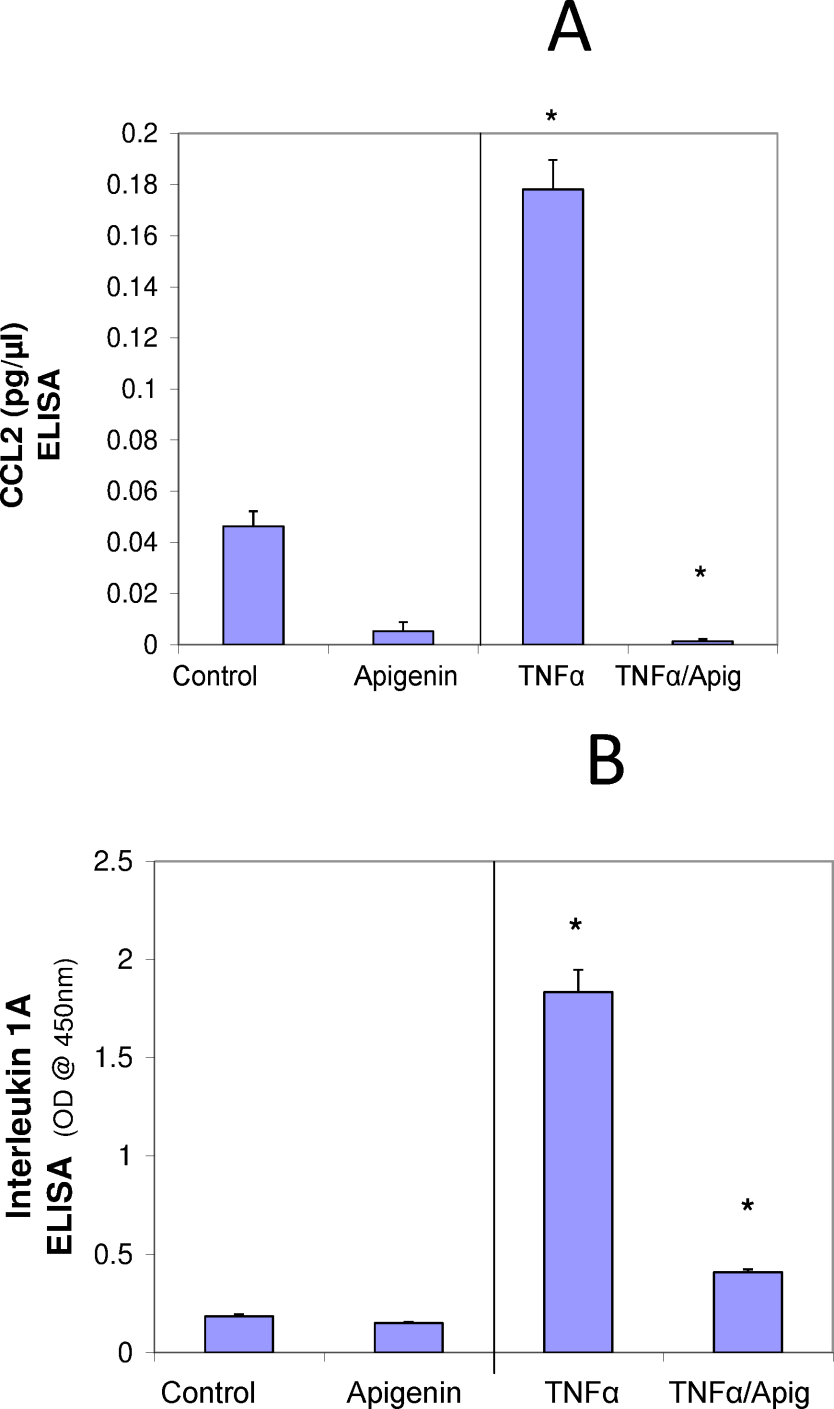
**Effect of Apigenin on TNFα mediated changes in CCL2 (A) and IL-1α (B) in MDA-MB-231 cells.** The data represent CCL (pg/ul) or IL-1α (O.D. @ 405nm) as Mean ± S.E.M. n = 3. The significance of differences between the Ctrl and TNFα groups and TNFα vs. TNFα + Apigenin were determined by a Students t-test *p<0.05.

A basic cell viability in MDA-MB-231 cells was performed to establish appropriate non-lethal working concentrations of apigenin and TNFα (Fig 1A) and (Fig 1B), respectively. Based on these baseline studies, we chose the concentrations of 40μM of apigenin and 40ng/ml of TNFα to carry out all subsequent studies. A semiquantitative analysis was performed to study the cytokine release pattern, in the presence of TNFα and +/- apigenin, using sandwich-based protein arrays from RayBiotech (Norcross, GA, USA). The data obtained show that TNFα caused significant upregulation of few specific cytokines. These cytokines included GM-CSF, CCL2, IL-1α, IL-6 (Fig 2A–2D) IL-8 and GRO CXCL1/CXCL2/CXCL3 a/b/g (Fig 3A–3D), some of which were downregulated by apigenin and subject to further validation by ELISA assays. First, the densitometry array values (reflected by INT/MM2) are shown for the effect of apigenin on TNFα mediated release of GM-CSF (Fig 4A), IL-6 (Fig 4B), CCL2 and IL-1α (Fig 4C). Results were then validated by independent ELISA, where the data paralleled between the findings in the arrays for both CCL2 and IL-1α (Fig 5A and 5B).

**Fig 6:**
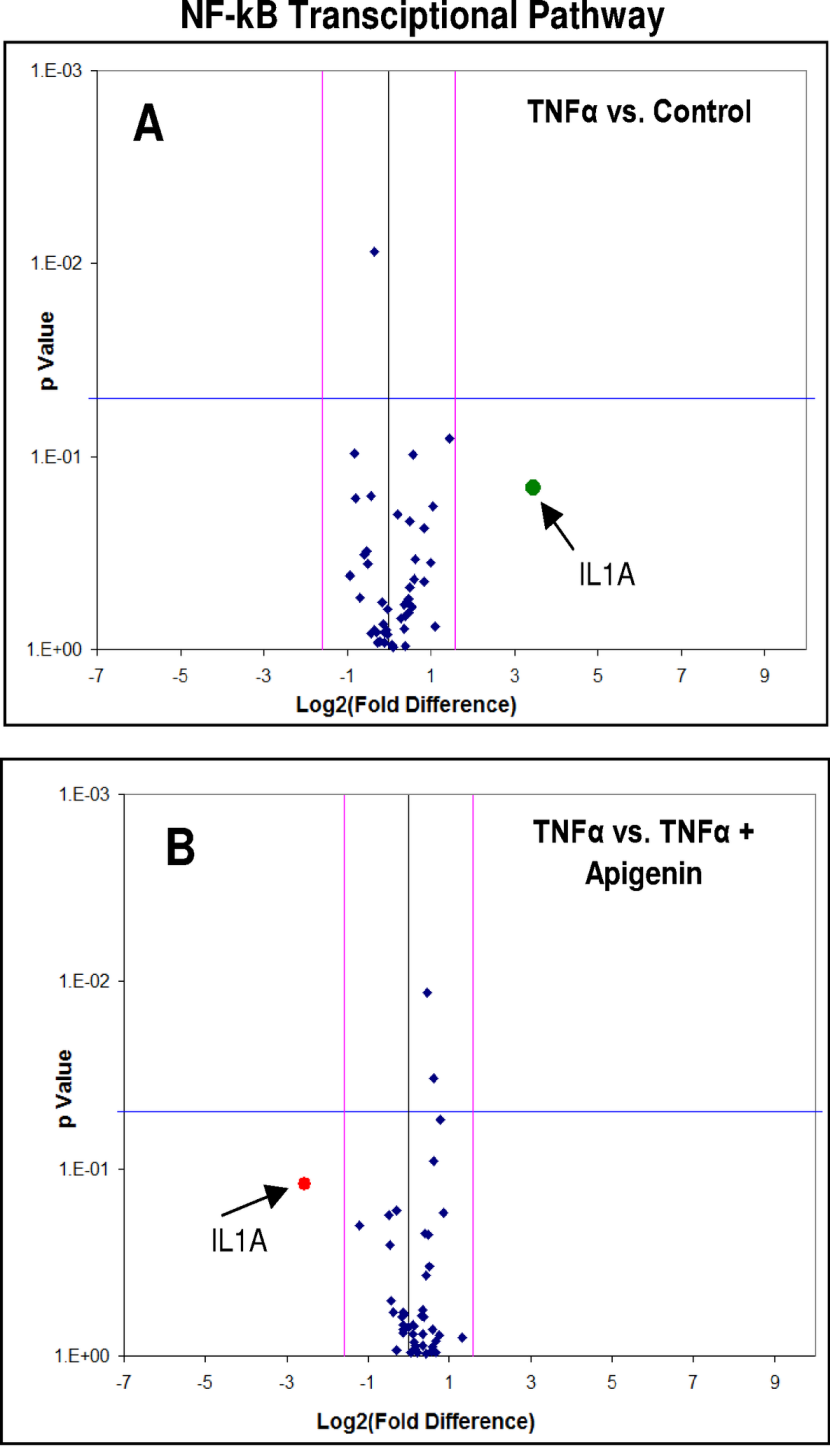
NF-kB PCR microarray assessment on MDA-MB-231 cells ± TNFα ± Apigenin. The data displays differential transcription for controls vs. TNFα (A) and TNFα vs. TNFα + Apigenin (B) in MDA-MB-231 cells treated for 24 hours. The data show no significant changes at P < .05 for either analysis, with non-significant differences for only IL-1α.

**Fig 7:**
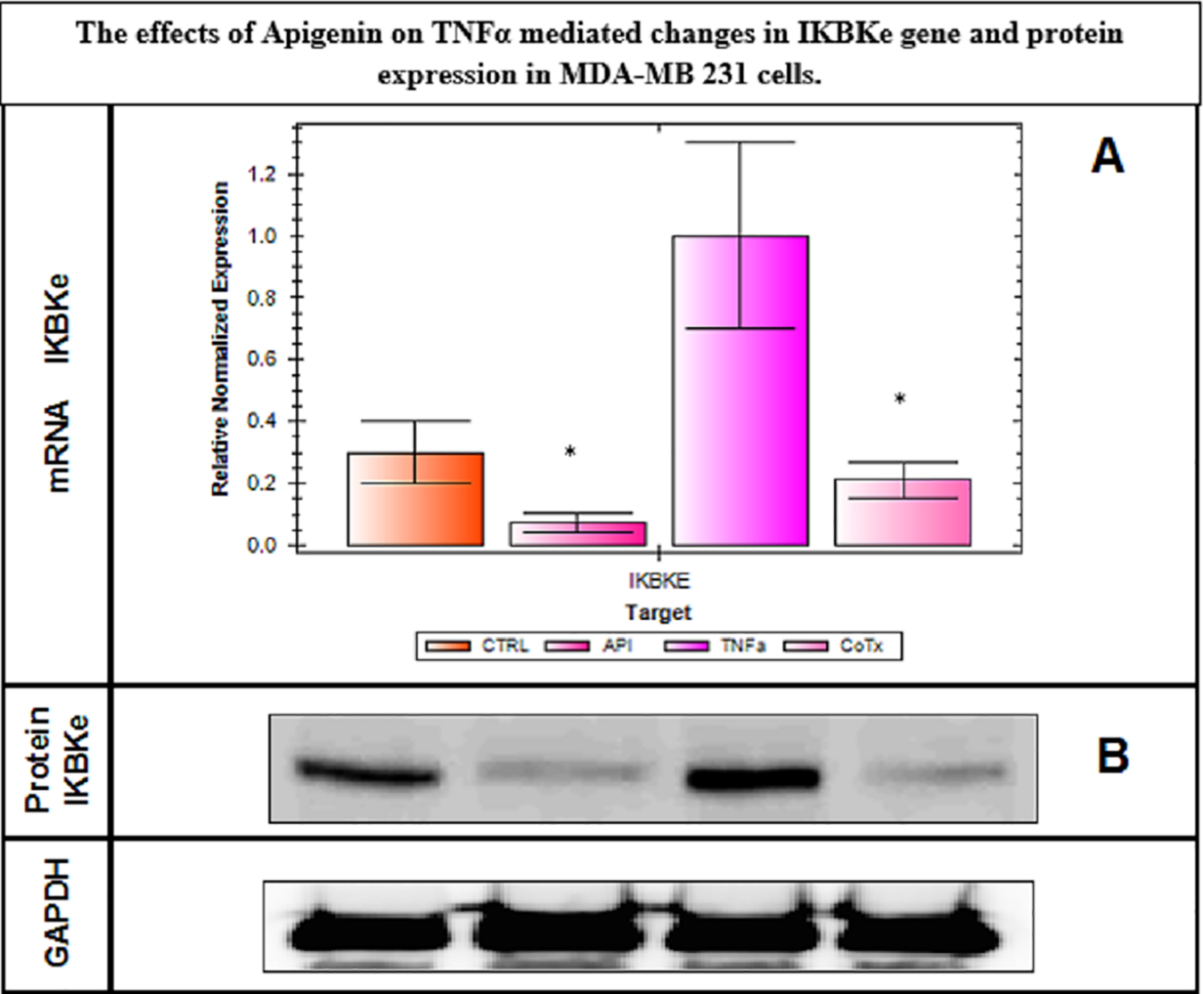
**A. IKBKe transcription in MDA-MB-231 cells ± TNFα ± Apigenin.** The data represent normalized expression and are expressed as the Mean ± S.E.M., n = 3. The significance of differences between the Ctrl and TNFα groups and TNFα vs. TNFα + Apigenin were determined by a Students t-test *p<0.05. **Figure 7B.** Protein expression of **IKBKe**/GAPDH with TNFα ± Apigenin at 24 hours.

**Fig 8:**
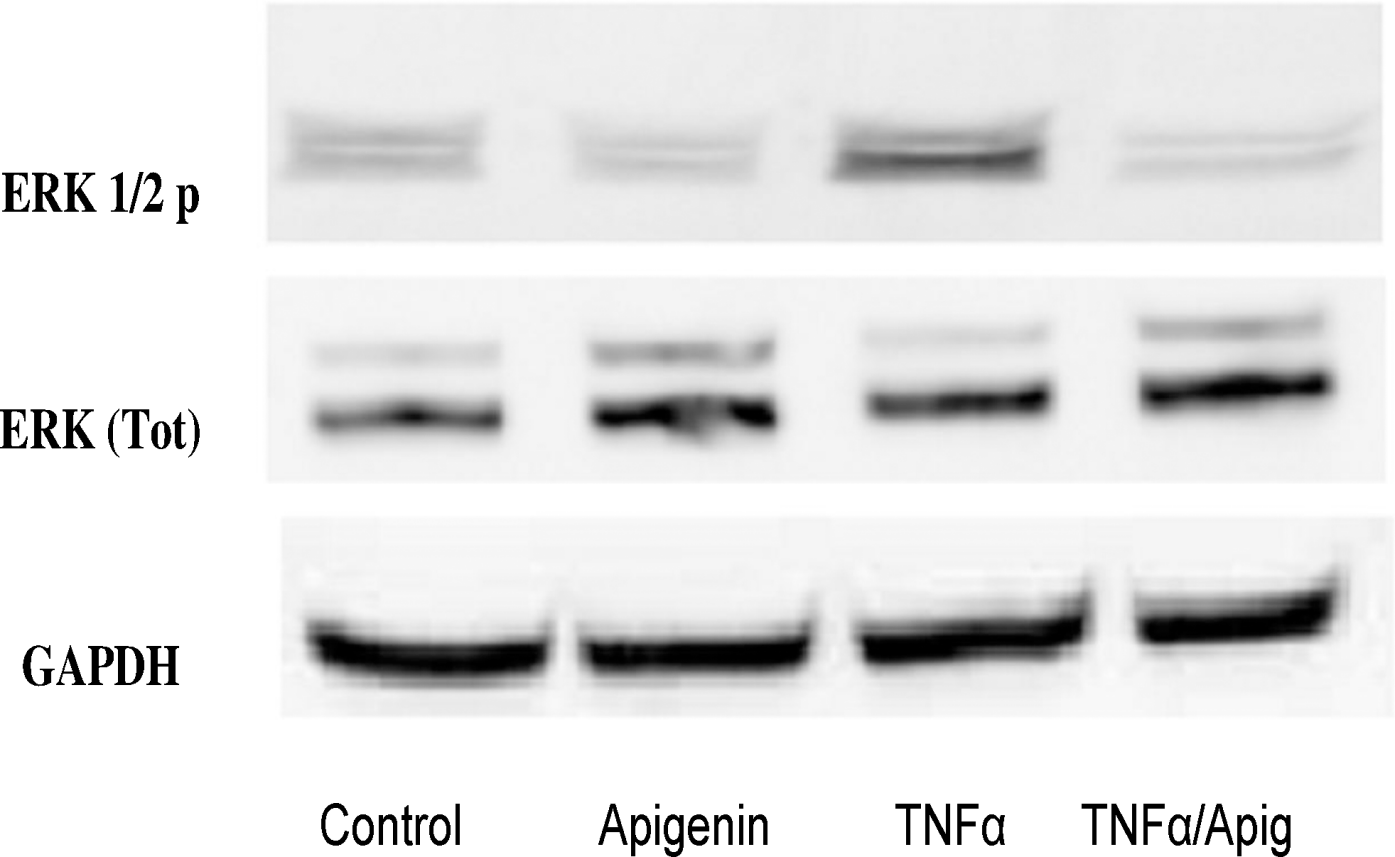
Protein expression of ERK 1 2 (total and phosphorylated) and GAPDH in MDA-MB-231 cells ± TNFα ± Apigenin.

To elucidate potential signaling changes associated with these findings, which were occurring at the transcriptome level, RT-PCR NF-κB arrays were conducted using Signaling Pathway H96 Predesigned 96-well panel for use with SYBR® Green.CAT Number: 10025558. Biorad (Hercules, CA, USA). The data show a lack of significant changes in all mRNAs involved with NF-κB transcriptome pathway, where IL-1α shows a distinct but non-significant change (Fig 6A and 6B and Table 1). The lack of data as reflected by this RT-PCR NF-κB array was surprising, but a limitation to this array was the absence of IKBK-epsilon which plays a pivotal role in driving tumor malignancy in both TNBC [39] and receptor positive breast cancer cells [40]. Therefore, in the next study, we investigate changes in IKBK-epsilon transcription determined by RT-PCR (Fig 7A) and protein expression using Western blot (Fig 7B). These findings validate IKBK-epsilon to be the major proponent of NF-κB signaling control involved with TNF-α mediated rise in CCL2 release and its reduction by apigenin. In the next study, we demonstrate additional influential effects of MAPK signaling on TNF-α mediated CCL2 release, focusing on changes in phospho-ERK protein, also downregulated by apigenin (Fig 8). These findings show that apigenin can alter TNF-α mediated breast cancer signaling pathways that control the release of CCL2, which in part involve IKBK-epsilon, and ERK, both essential biological events required for TAM infiltration and recruitment into solid breast tumor tissue.

**Table 1:** NF-kB PCR microarray tabulated data on MDA-MB-231 cells ± TNFα ± Apigenin. The data displays differential transcription as Log2 (Fc) for controls vs. TNFα (Left Panel) and TNFα vs. TNFα + Apigenin (Right Panel) in MDA-MB-231 cells treated for 24 hours. The data show no significant changes at P < .05 for either analysis, with non-significant differences for only IL-1α.

TNF-alpha vs Controls			TNF-alpha vs TNF-alpha+Apigenin		
Symbol	Log2(Fc)	p Value	Symbol	Log2(Fc)	p Value
IL1A	3.47	0.14	IL1A	-2.555	0.121
AKT1	-0.03	0.83	AKT1	0.088	0.769
AKT2	-0.36	0.01	AKT2	-0.122	0.6
AKT3	-0.27	0.92	AKT3	0.415	0.975
CD14	-0.8	0.17	CD14	-0.482	0.176
CD28	-0.29	0.81	CD28	0.168	0.923
CHUK	0.27	0.69	CHUK	0.655	0.833
IKBKB	-0.07	0.79	IKBKB	0.851	0.173
IKBKG	0.84	0.24	IKBKG	0.375	0.619
IL1R1	0.46	0.55	IL1R1	-0.019	0.705
IL1RAP	0.35	0.58	IL1RAP	-0.169	0.616
IRAK1	-0.6	0.33	IRAK1	-0.445	0.507
IRAK2	0.6	0.43	IRAK2	0.515	0.334
IRAK3	0.98	0.35	IRAK3	-1.222	0.201
LBP	-0.29	0.81	LBP	0.168	0.923
LTA	1.12	0.76	LTA	0.488	0.225
LTBR	0.63	0.34	LTBR	-0.372	0.584
LY96	0.52	0.6	LY96	0.755	0.776
MAP3K1	-0.44	0.82	MAP3K1	0.138	0.846
MYD88	-0.52	0.36	MYD88	0.421	0.371
NKKB1	0.45	0.65	NKKB1	0.328	0.609
NFKB2	0.57	0.1	NFKB2	0.778	0.055
NFKBIA	1.04	0.18	NFKBIA	0.105	0.687
NFKBIB	0.48	0.22	NFKBIB	0.625	0.092
NFKBIE	0.84	0.45	NFKBIE	0.348	0.656
PRKCQ	-0.29	0.81	PRKCQ	0.168	0.923
REL	0.53	0.6	REL	0.665	0.956
RELA	-0.43	0.16	RELA	0.605	0.033
RELB	1.45	0.08	RELB	0.395	0.222
RIPK2	0.38	0.67	RIPK2	0.598	0.72
SUMO1	0.06	0.95	SUMO1	-0.299	0.927
TBP	-0.13	0.74	TBP	0.558	0.946
TLR4	0.09	0.98	TLR4	-0.149	0.587
TNF	0.37	0.95	TNF	0.215	0.955
TNFRSF1A	-0.16	0.57	TNFRSF1A	0.118	0.692
TNFRSF1B	0.49	0.47	TNFRSF1B	-0.465	0.255
TRADD	-0.54	0.31	TRADD	0.045	0.959
TRAF2	0.2	0.2	TRAF2	0.438	0.012
TRAF6	0.35	0.78	TRAF6	-0.129	0.728
UBB	-0.82	0.1	UBB	-0.295	0.167
UBC	-0.12	0.92	UBC	-0.135	0.685

## Discussion

The findings in this study describe another anti-cancer property of apigenin to add to the arsenal of existing research having shown its ability to attenuate tumor promoting enzymes [1,2], prevent chemoresistance/multi-drug resistance [3–5], halt angiogenesis [6] lessening VEGF, inhibiting aromatase, proteasomal processes and fatty acid synthase, [18–21] and an overall reduction in proliferation, migration, and invasion of aggressive breast cancer [7]. Here we demonstrate the ability of apigenin to block TNFα mediated release of pro-inflammatory cytokines which are themselves responsible for inward migration of leukocytes that drive tumor growth, metastatic invasion [27], stem cell survival and immune evasion [23].

TNFα evoked release of tumor-promoting chemokines such as CCL2 (MCP-1), CCL5 (RANTES) and CXCL8 (IL-8) [26] trigger infiltration of CCR2/CCR5 receptor bearing leukocyte sub-populations (LSPs) including TAMs [29], MDSCs [30], TANs[31,32], Tregs [33], MAMs and CAFs [34]. While all LSP subtypes enable a tumor permissive and the evasive immune environment [41], independently, each LSP can confer a unique independent effect. For example, the arrival of MDSCs leads to potent immunosuppression by disabling the normal function of T and NK cells [42]. Whereas, the arrival of TANS perpetuates a sustained release of CCL2, GM-CSF, CCL17 which correspond to elevated tumor size, microvascular invasion and inward migratory activity of the macrophages and Tregs [43]. Reported results on Tregs indicate a direct role in suppression of antitumor immunity by attenuating the T-cell-mediated tumor cell killing [33] and secreting TGF-beta1 [44,45]. Meanwhile, the collective activity of TANS and MDSCS both yield the perpetual release of CCL2 and M-CSF fostering synergistic infiltration of TAMS. TAMS, upon arrival, can then lead to even more release of promoting cytokines: TGF-beta, MMPs, VEGF, CM/M-CSF, CCL 17, IL-13 and CCL2 which secure pro-angiogenic tumor processes [46]. In aggressive malignancies, metastatic MAMs arrive at secondary sites and in turn recruit inflammatory CCR2 bearing monocytes by CCL2, which in themselves release CCL3 and express CCR1, amplifying tumor potential [47]. LSP tumor infiltration is a vastly complex topic, yet all of these studies suggest the importance of controlling the chemokines produced and released by tumor tissue.

The current study shows that apigenin can downregulate TNFα mediated release of CCL2, largely attributable to its ability to downregulate IKBKe. The importance of IKBKe in driving tumor malignancy potential in both TNBC [39] and receptor positive breast cancer cells are known [40]. Moreover, there is a correlation between concentration of IKBKe and activated JAK/SAT[39] TRAF2 [48] Akt/PI3K [49] phosphorylated p65-NFkB [40] and NF-kB activation [50]. Likewise, numerous studies show many of these signaling pathways are blocked by apigenin in tumor models [17] in particular NF-kB signaling [51,52] which controls the downstream release of TGF-b [53] and activity of MMP-9 [54], themselves involved with TAM/TANs [55–57] and Treg recruitment [44,45].

The capacity of apigenin to block CCL2 is important because this single event is not only in control of infiltrating/migratory activity of CAFs, TAMS, TANS, and MSCs into the tumor environment but enables its perpetual ongoing accelerated release of CCL2, CXCL8, CCL5, mediated by through NF-kB signaling [24]. There is sufficient evidence to show that initial release of chemokines such as CCL2, GM-CSF [58] mark a potentially irreversible turning point for tumor infiltration, immunological evasion, and tumor immune support [29,59,60]. Natural plant-derived chemicals that can downregulate CCL2 or act as CCR2 receptor antagonists [61] such as luteolin [62] esculetin [63] or EGCG [64,65] typically result in impaired migration, less proclivity for metastasis [66] enabling greater efficacy of immunotherapies and chemotherapy drugs [65].

In summary, these findings show that apigenin can block TNFα mediated release of CCL2 in a TNBC cell line. While the experimental evidence for the therapeutic application of apigenin in cancer treatment is growing, human clinical trials are lacking [67]. Future studies will be required to determine if apigenin can be used clinically to establish long-term remission in cancer patients.

## Materials and methods

### Cell line, chemicals, and reagents

Triple-negative human breast tumor (MDA-MB-231) cells were obtained from the American Type Culture Collection (Rockville, MD, USA). Dulbecco’s modified Eagle’s medium (DMEM), fetal bovine serum (FBS) and penicillin/streptomycin were all obtained from Invitrogen (Carlsbad, CA, USA). Recombinant human TNFα was purchased from RayBiotech (RayBiotech Inc., Norcross, GA, USA). Apigenin was purchased from Abcam (Cambridge, United Kingdom).

### Cell culture

MDA-MB-231 cells were cultured in 75 cm2 or 175 cm2 flasks containing DMEM supplemented with 10% FBS and 1% 10,000 U/ml penicillin G sodium/10,000 μg/ml streptomycin sulfate. Cells were grown at 37°C with humidified 95% air and 5% CO2.

### Cell viability assay

Alamar Blue cell viability assay was used to determine cytotoxicity. Viable cells are capable of reducing resazurin to resorufin, resulting in fluorescence changes. Briefly, 96-well plates were seeded with MDA-MB-231 cells at a density of 5×104cells/100 μl/well. Cells were treated without or with either apigenin (10 μM, 20 μM, 30 μM, 40 μM 50 uM, 60 μM 70 μM 80 μM 90 μM 100 μM) or TNFα (0.1, 1, 10, 20, 40, 80, 100 ng/ml) for 24 h at 37°C, 5% CO2. Alamar blue (0.1 mg/ml in HBSS) was added at 15% v/v to each well and incubated for 6–8 hrs. Quantitative analysis of dye conversion was measured on a microplate fluorometer–Model 7620-version 5.02 (Cambridge Technologies Inc, Watertown, MA, USA) set at 550/580 (excitation/ emission). The data were expressed as a percentage of live untreated controls.

### Human adipokine array

Sandwich-based arrays purchased from RayBiotech (Norcross, GA, USA) consist of membranes with 62 different proteins in duplicate. Each experiment was carried out by manufacturer’s instructions. Briefly, antibody-coated array membranes were treated with 1 ml of medium from resting, apigenin-treated (40 μM), TNFα-treated (40 ng) and co-treated cells and incubated overnight at 4°C on a rocker/shaker. The medium was decanted, the membranes were washed with wash buffer and then incubated with 1 ml biotin-conjugated antibodies (overnight 4°C). The mixture of biotin-conjugated antibodies was removed, and membranes were incubated with horseradish peroxidase–conjugated streptavidin (2 h). After a final wash, membrane intensity was acquired using chemiluminescence and analyzed using Quantity One software (Biorad Laboratories, Hercules. CA, US). Densities were calculated by subtracting blanks values from each array, then calculating all values as a percentage of the positive control spots on each membrane multiplied by an arbitrary averaged control values across all arrays in one individual test set. The data are represented as Density (Intensity (INT)/MM2).

### CCL2 and IL-1α detection by ELISA

Supernatants from resting and stimulated (24 h) MDA-MB-231 cells were collected and centrifuged at 100× *g* for 5 min at 4°C. Specific ELISAs were performed using MCP-1/ CCL2 ELISA kits (RayBiotech) following the manufacturer’s instructions. Briefly, 100 μl of supernatants from samples and standards were added to 96-well plates pre-coated with capture antibody. After incubation, 100 μl of prepared biotinylated antibody mixture was added to each well. After one hour, the mixture was decanted, and 100 μl streptavidin solution was placed in each well and incubated. Substrate reagent (100 μl) was then added to each well followed by the addition of 50 μl stop solution 30 min later. The plate was read at 450 nm using UV microplate reader.

### RT-PCR and RT-RCR NF-kB signaling pathway

MDA-MB-231 cells were lysed with 1 ml Trizol reagent. Chloroform (0.2 ml) was added samples were vortexed, incubated at 15–30°C for 2–3 min and centrifuged at 10,000 x g for 15 min. at 2–8°C. The aqueous phase was transferred to a fresh tube, and the RNA precipitated by mixing 0.5 ml isopropyl alcohol. RNA was extracted and subject to iScript advanced reverse transcriptase (RT) to the reaction. The reverse transcription was performed for 30 min at 42°C and RT inactivation for 5 min. at 85°C. PCR reaction. The following components were mixed in a 0.5 ml PCR tube: 5.0 μl cDNA product, 10 μl Ss Advanced Universal SYBR^®^Green, 1.0 μl primer and 4 μl water. PCR was performed with 39 cycles of denaturation: 15 sec. at 95°C; annealing: 30 sec. at 60°C; and extension 60 sec. at 72°C using Bio-Rad CFX96 Real-Time System (Hercules, Ca, USA). cDNA synthesis and Real-Time PCR was performed using iScript Advanced synthesis kit / SsAdvanced Universal SYBR^®^ Green according to manufacturer's instructions. RT-PCR for IKBKe was run normalized to GAPDH mRNA, and the normalized values for the NF-kB Signaling array are as specified in Fig 6A. The array used was the transcription—NF-kB Signaling Pathway H96 Predesigned 96-well panel for use with SYBR® Green (Bio-Rad, Hercules, CA).

### Western blot *ERK1/2 and IKBKe*

Total cell protein concentrations from MDA-MB-231 cells treated with apigenin, with and without TNFα co-treatment, was determined using a modified Bio-Rad “DC” protein assay (Bio-Rad Laboratories, Hercules, CA, USA). Cell lysates were separated by electrophoresis on 10% SDS-polyacrylamide gels and then transferred to Immobilon-P PVDF membranes. Blots were blocked at 4°C overnight in 5% bovine serum albumin (Sigma, St. Louis, MO, USA) in Tris-buffered saline with 0.05% Tween 20 in PBS (PBST) and then incubated overnight at 4°C with mouse anti-human ERK1/2 and **IKBKe** affinity purified antibody (Cell Signaling, Danvers, Ma, USA). Membranes were washed with PBST and incubated overnight with anti-goat IgG-horseradish peroxidase (Santa Cruz Biotechnology, CA) in PBST overnight at 4°C. Protein loading was monitored in each gel lane by probing the membranes with anti-GAPDH antibodies (Santa Cruz, Ca, USA). Immunoblot images were obtained using a Flour-S Max Multimager (Bio-Rad Laboratories, Hercules, CA). Lane density data was acquired with Quantity One Software (Bio-Rad Laboratories, Hercules, and CA).

### Statistical analysis

Statistical analysis was performed using GraphPad Prism (version 3.0; GraphPad Software Inc. San Diego, CA, USA) with the significance of the difference between the groups assessed using a one-way ANOVA, followed by Tukey *post hoc* means comparison test, two-way ANOVA or Student’s *t*-test.
